# Active viral infection during blooms of a dinoflagellate indicates dinoflagellate-viral co-adaptation

**DOI:** 10.1128/aem.01156-23

**Published:** 2023-10-24

**Authors:** Jingtian Wang, Ling Li, Senjie Lin

**Affiliations:** 1 State Key Laboratory of Marine Environmental Science, College of the Environment and Ecology, and College of Ocean and Earth Sciences, Xiamen University, Xiamen, China; 2 Department of Marine Sciences, University of Connecticut, Groton, Connecticut, USA; Colorado School of Mines, Golden, Colorado, USA

**Keywords:** dinoflagellates, virus, bloom, organelle, infection

## Abstract

Viruses are generally believed to cause cell mortality and terminate algal blooms. However, how the dinoflagellate-virus interaction shapes the dynamics of host dinoflagellate blooms remains poorly understood. Here, we profile viral composition and metabolic landscape in two blooms of the widely distributed dinoflagellate *Prorocentrum shikokuense*. Our data show that *P. shikokuense* was infected dominantly by *Mimiviridae* and *Phycodnaviridae* viruses in both blooms. Strikingly, these viruses were transcriptionally very active during the bloom that extended for weeks, suggesting a paralleled development of both the bloom and chronic infection. Furthermore, our data indicate that these viruses maintained a stable infection process by generating polymorphic variants to sustain the exploitation of host intracellular machinery, suggesting co-adaptation between the viruses and the bloom-causative dinoflagellate. This novel insight will be valuable for fully understanding and modeling the role of viruses in regulating blooms of dinoflagellates and other algae.

**IMPORTANCE:**

This study represents the first that investigates *in situ* virus infection in dinoflagellate blooms. Our findings reveal highly similar viral assemblages that infected the bloom species *Prorocentrum shikokuense* and a co-adapted metabolic relationship between the host and the viruses in the blooms, which varied between the prolonged and the short-lived blooms of the same dinoflagellate species. These findings fill the gap in knowledge regarding the identity and behavior of viruses in a dinoflagellate bloom and shed light on what appears to be the complex mode of infection. The novel insight will be potentially valuable for fully understanding and modeling the role of viruses in regulating blooms of dinoflagellates and other algae.

## INTRODUCTION

Marine phytoplankton not only acts as primary producers in the food chain but also plays an essential role in the global carbon cycle and geochemical processes in the ocean. However, under certain conditions, some species’ explosive proliferation and aggregation or toxin production lead to harmful algal blooms (HABs), which cause devastating impacts on the ecosystem, economy, and human health. HABs can last for weeks and collapse within a few days under specific environmental factors. Viruses, the most abundant living entity in the ocean, are recognized as a crucial factor contributing to algal mortality and the dynamics of HABs (1 – 6).

However, increasing evidence from non-dinoflagellate phytoplankton shows that viral infection may not necessarily cause host algal cell lysis, and rather, viruses and hosts can coexist under certain conditions. For instance, the haptophyte *Phaeocystis pouchetii* can coexist with its virus PpV-01 for 40 days after infection (7). The chlorophyte *Chlorella variabilis* and its virus PBCV-1 was observed to maintain a 15-day stable interaction following a 30-day arms race battle (8). The dynamics of the prasinophyte *Ostreococcus* and its infective double-stranded DNA (dsDNA) virus OmV2 were found to grow in parallel (9). The lytic viruses of the haptophyte *Emiliania huxleyi* (EhVs) can adopt a chronic infection strategy at the early stage of infection and transmit horizontally through budding without causing cell lysis, also known as pseudolysogeny or carrier states (3, 10 – 12). Intriguingly, the prolonged virus-host coexistence can be achieved through periodic rapid minor genetic changes in the mode of single-nucleotide polymorphisms (SNP) (13, 14). These behaviors are characteristic of Red Queen dynamics, which posits that biotic interactions drive molecular evolution such that rapid genotypic changes within the population arise from ecological and evolutionary mechanisms (13, 15 – 17). This implies an evolutionary arms race between the virus and its host, in which the dynamic internal genotype changes maintain the apparent stability of the virus-host coexistence.

However, the mode of interaction between viruses and harmful algae and the role of the infecting viruses in regulating the dynamics of harmful algal blooms are still poorly understood and underexplored. In particular, whether the chronic mode of viral infection occurs in dinoflagellates, the most dominant causative species of HABs, and if so, what mechanism drives the long-term virus-host coexistence in bloom outbreaks remains elusive. This study was based on time-sequential metatranscriptomics of two bloom outbreaks by *Prorocentrum shikokuense* (one prolonged and the other short-lived), and it aimed to characterize the interaction between viral infection and algal bloom progression. We identified the viruses infecting *P. shikokuense* during the blooms and found the co-evolution of their infection mode with the host bloom. We found that chronic infection was the main mode in the prolonged bloom. Furthermore, our data indicated that in the long-duration bloom, viruses might maintain the stability of functional expression through genetic polymorphism to sustain the utilization of host organelles and ensure stable chronic infection.

## RESULTS

### Dominance of *Mimiviridae* and *Phycodnaviridae* genes expressed during the blooms

**Fig 1:**
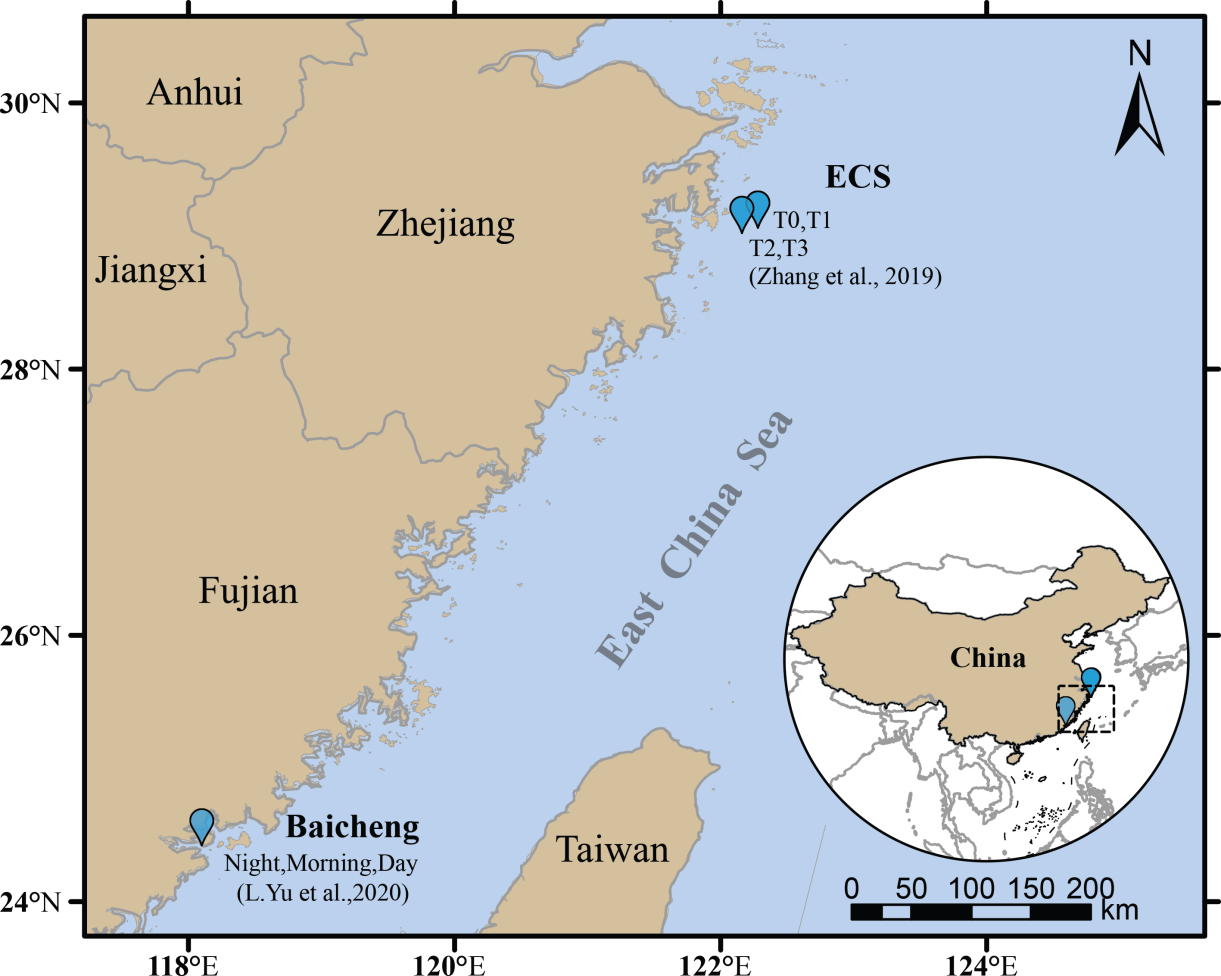
Sampling sites of this study: East China Sea (ECS) and Baicheng (BC).

Plankton samples were collected during two dinoflagellate blooms in 2014 at two locations (Baicheng, BC and East China Sea, ECS) that were 900 km apart (Fig. 1). Previous studies’ microscopic sequencing and RNA sequencing confirm that *P. shikokuense* is the dominant species in BC and ECS, accounting for 76% and 74.2%, respectively (18, 19). Pooling transcriptomes and clustering predicted protein sequences yielded 659,984 unique genes in the bloom at BC and 553,342 unique genes in the bloom at ECS; other assembly and annotation information is listed in Table S1.

**Fig 2:**
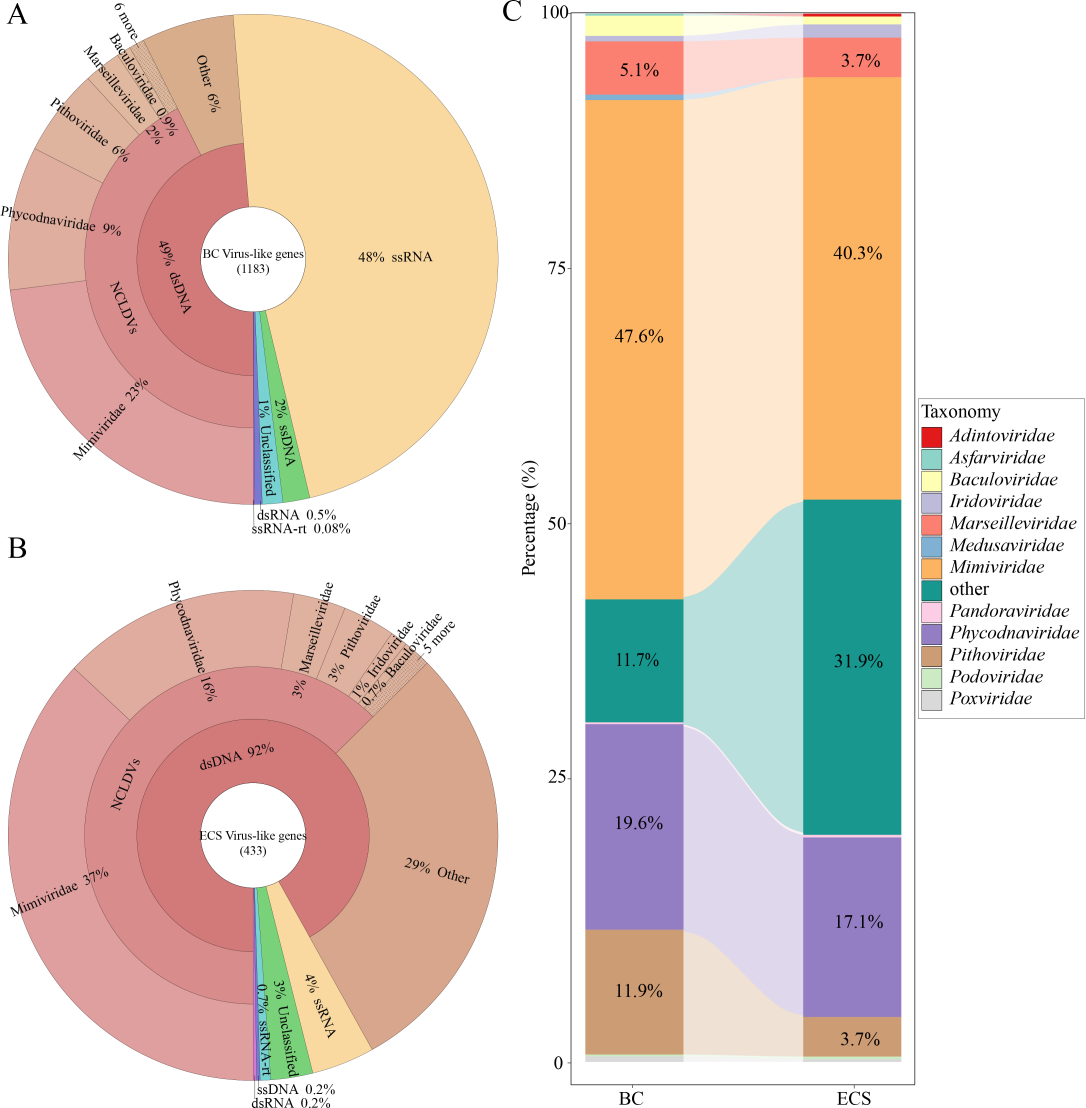
Virus-like genes statistics and taxonomic representation in BC (**A**) and ECS (**B**) blooms of *P. shikokuense*. The number in the center of the circles (**A and B**) depicts the total number of virus-like genes. (**C**) Comparison of dsDNA viral taxonomic profile represented in the virus-like gene sets in BC and ECS blooms. A number of unigenes with the nearest BLAST matches were used to calculate the proportion. (Unique CDSs were determined using a cut-off of <95% pairwise sequence identity.)

The metatranscriptomes revealed distinct algae-associated viral communities in the blooms at BC and ECS. The total number of virus genes in BC (1,183 genes) was more than twice than that in ECS (433 genes). The more abundantly expressed viral genes in BC belong to double-stranded DNA (dsDNA) and single-stranded RNA (ssRNA) viruses, accounting for 49% and 48%, respectively (Fig. 2A and B), whereas the expressed viral genes in the ECS bloom were predominantly originated from dsDNA virus (92%), with ssRNA and other types of viruses together accounting for 8%. Further examination revealed that a substantial proportion of the ssRNA-virus genes in the BC bloom matched viruses of mammals and invertebrates (Fig. S1). Considering the close proximity of the BC bloom to land and human activity (11 km from the nearest land, compared to 450 km for ECS from the nearest land), these mammalians and invertebrate ssRNA viruses-like viruses in BC were likely terrestrially derived contaminants adsorbed to particles. In coastal and estuarine environments, suspended particles in the water column facilitate the survival of viruses (20), urbanizing and agricultural activities may promote viral diversity, and land use can affect viral communities (21). Therefore, the ssRNA-virus-associated data were excluded from the subsequent analysis.

The remaining dsDNA virus data showed striking similarity between the ECS and BC blooms. The majority of dsDNA type virus-like genes belonged to families *Mimiviridae* and *Phycodnaviridae* in both blooms (Fig. 2C; Table S2). Although the numbers of unigenes (clusters of genes with >95% sequence similarity) corresponding to the *Mimiviridae* and *Phycodnaviridae* were notably higher in the BC bloom (272 and 112 unigenes, respectively) than in the ECS bloom (160 and 68, respectively), their proportions in the total dsDNA virus-like genes were highly similar (47.6% versus 19.6% in BC compared to 40.3% versus 17.1% in ECS).

For natural bloom communities, the cell (particle)-associated viruses detected theoretically could be viruses infecting non-bloomed algae, protists, and nonliving particles. To determine if these *Mimiviridae* and *Phycodnaviridae* we detected were associated with *P. shikokuense*, we compared the bloom-derived gene transcripts with transcripts from laboratory-cultured *P. shikokuense* (22) using BLAST analysis. Except for *Marseilleviridae*, the top five taxa of viruses from the cultures matched those from BC and ECS bloom samples (Fig. S2). This result suggests that the dominant viral genes expressed in the two blooms were viruses that infected *P. shikokuense*.

### Upregulated expression of viral genes during bloom relative to non-bloom

**Fig 3:**
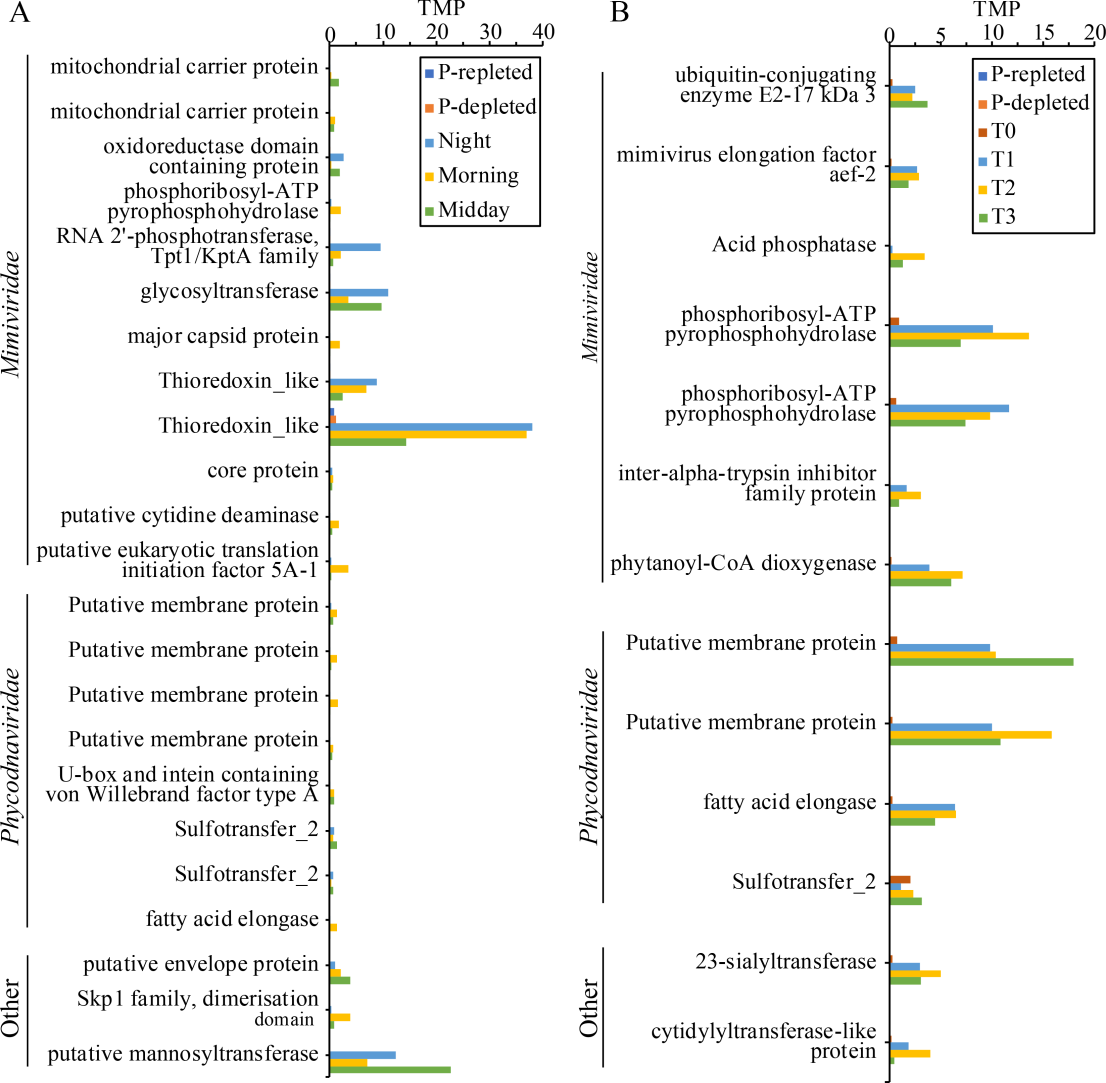
Putative viral genes from ECS (**A**) and BC (**B**) that showed a greater than twofold elevated or exclusive expression in the blooms relative to non-bloom or culture samples. TPM: transcripts per million.

Of the total viral genes, 3% (13 unigenes) and 4% (23 unigenes) showed elevated [twofold transcript per million (TPM) change] expression or exclusive expression in the ECS and BC bloom relative to the non-bloom (T0) field samples or laboratory culture (Fig. 3; Table S3). The expression levels of the upregulated viral genes in the two blooms were as high as >35 TPM. Based on the functional annotation, most genes are involved in early stages of viral infection. Specifically, most of the 23 upregulated genes annotated in BC are involved in virus contact entry and genome replication (Fig. 3A). These included four membrane proteins, with attachment and entry functions to facilitate the attachment of viruses to the host cell and promote fusion between viral and host cell membranes (23). An envelope protein also recognizes and binds to receptor sites on the host’s membrane for membrane fusion (attachment and entry) (24). Glycosyltransferase and mannosyltransferase are responsible for glycosylating viral proteins during virus replication (genome replication) (25). The thioredoxin-like gene was the most highly expressed in the BC bloom, and it is known to regulate viral replication by inhibiting antiviral signal transduction (genome replication) (26). We also identified two mitochondrial carrier proteins, which may function in exploiting the mitochondrion of its host by harvesting its dATP and dTTP for genome replication (27). Also in this group of proteins was cytidine deaminase, which causes G to A mutation in the complementary strand during DNA replication (genome replication) (28), and major capsid protein or *mcp* (genome assembly and release), which is usually expressed in the late stage of the viral infection, responsible for synthesizing virus capsid and participating in the assembly of new virions (29, 30).

Similar to BC, most of the 13 upregulated viral genes in the ECS bloom function in the early stage of virus infection (Fig. 3B). One of these codes for phosphoribosyl-ATP pyrophosphohydrolase, which may mediate the fusion between the virus envelope and the endosomal membrane (31, 32). Sulfotransferase assists multiple viruses for cell attachment or binding (attachment and entry) (33). Cytidylyltransferase-like protein is involved in synthesizing an essential core component of the lipopolysaccharide layer, suggesting a role in viral glycoprotein biosynthesis related to virion-cell recognition (attachment and entry) (34). Acid phosphatase may be necessary for regulating the fusion step of virus entry (attachment and entry) (35). E2 ubiquitin-conjugating enzyme is a virus replication protein involved in viral genome replication (36, 37). Elongation factor is essential for virus replication in infected host cells (38).

### Upregulated expression of host genes related to viral infection and anti-virus defense in blooms

**Fig 4:**
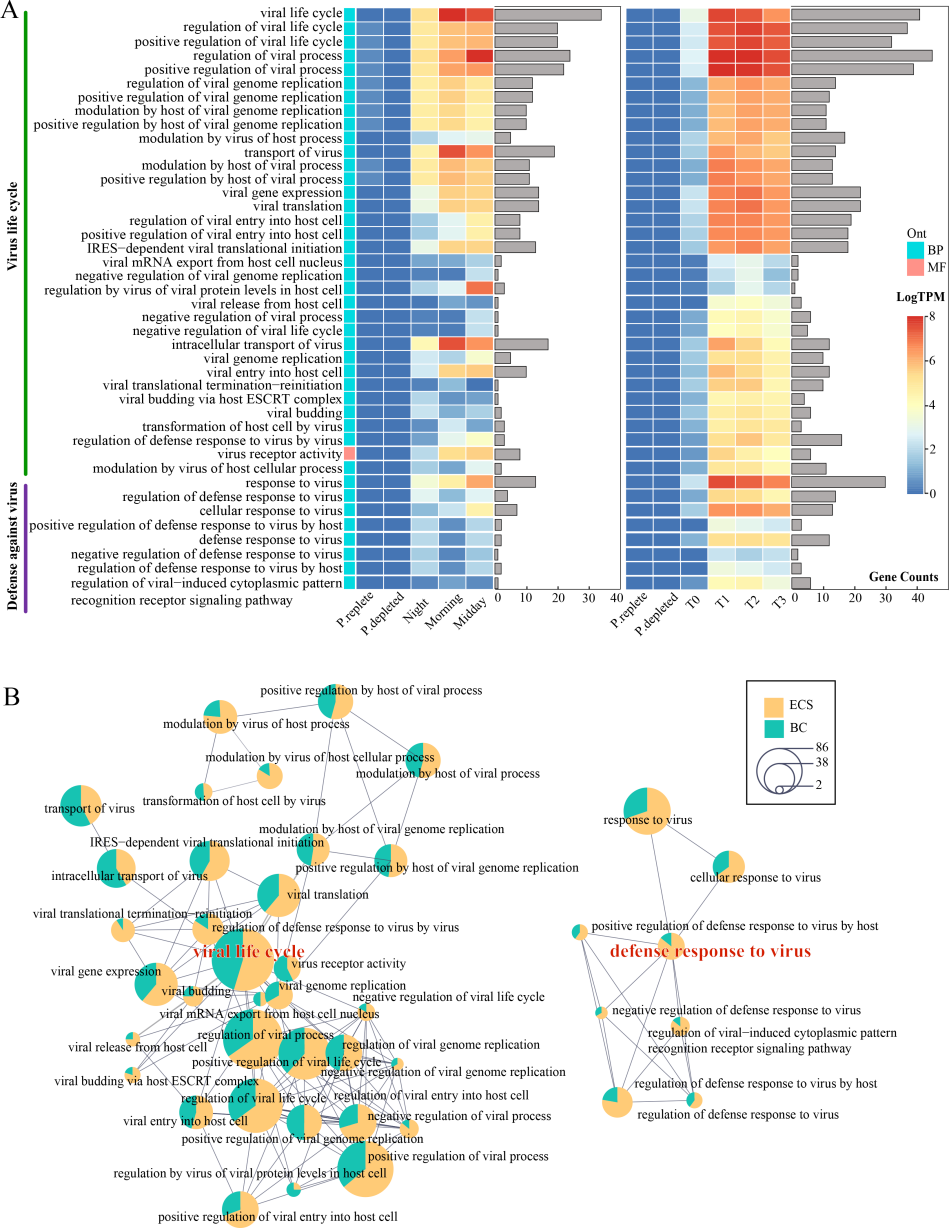
Common host-originated virus-related GO terms enriched by upregulated genes in BC and ECS blooms. (**A**) Expression heatmap and gene number histogram of common virus-related GO terms. (**B**) Enrichment map for common GO terms based on 42 virus-related GO terms. The GO terms of core functions are marked red, and the links indicate overlapping gene sets; the two parts of the pie chart represent the number of genes related to this GO term in ECS and BC, respectively.

We investigated how host cells (*P. shikokuense*) responded to, or were influenced by, the infecting viruses. Genes of *P. shikokuense* and other major lineages of phytoplankton (*Karlodinium* in ECS, *Syndiniales*, *Gonyaulacales,* and *Mediophyceae* in BC) (18, 19) were first separated based on NCBI species annotation. From these sorted data, we identified 81 Gene Ontology (GO) terms related to viral interactions, terms (including molecular function, cellular component, and biological processes) that unify the representation of gene and gene product attributes (39, 40). In these virus-related GO terms, only 6 GO terms (each with no more than 10 genes) were found in other algal lineages than *P. shikokuense* (Fig. S3), indicating that *P. shikokuense* was the primary host of these viruses in the blooms. Virus-related GO terms were then screened by different upregulated genes in BC and ECS belonging to *P. shikokuense* (Fig. S4). We found 42 common virus-related GO terms (Fig. 4A) shared by the two blooms. The most gene-rich GO term was functionally annotated as regulation of viral process (45 genes) in ECS and viral life cycle (34 genes) in BC (Fig. 4A).

All the host-originated virus-related GO terms were clustered into two major life activity processes: virus life activity and defense against virus, which was confirmed by the GO enrichment map (over 80% of GO terms related to virus life cycle) (Fig. 4B). Moreover, we found that the viral life cycle process was more active than the anti-virus defense in terms of gene number and expression level, particularly in ECS. The viral cycle-related genes that were upregulated in both blooms were functionally involved in the stage of viral infection subsequent to entry into the host, including genes involved in viral intracellular transport, viral replication, and transcription. However, the host’s expression of anti-virus defense GO terms decreased during the bloom in ECS when viral activity GO terms increased. In contrast, anti-virus defense GO terms in the BC bloom exhibited an increase compared to laboratory culture.

### Temporal expression dynamics of host photosynthesis and viral infection genes consistent with *P. shikokuense* abundance changes

**Fig 5:**
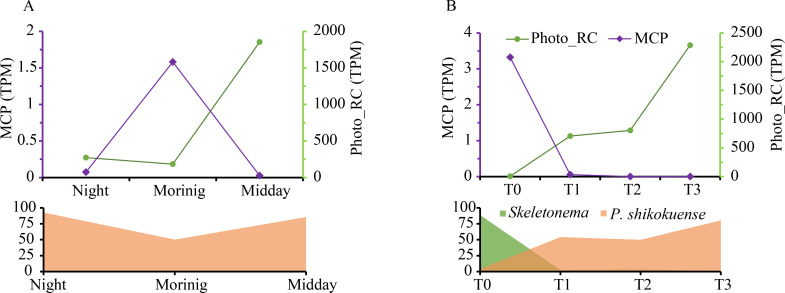
Opposing trends of *P. shikokuense* abundance and *psbA* gene expression versus *mcp* expression in BC (**A**) and ECS (**B**) blooms. The area plots at the bottom depict the proportion of the *P. shikokuense* population in the plankton community.

To track the effects of virus infection on the metabolic state of *P. shikokuense* during blooms, we adopted Vincent’s method (3) to use *psbA* and *mcp* genes as proxies of host photosynthesis and virus replication, respectively. We monitored the average transcript (mRNA) abundance of *psbA* in each sample. *psbA* is a chloroplast gene that encodes D1 protein, which is constantly transcribed in algal cells due to its rapid turnover (3, 41). Furthermore, we followed the expression of the viral *mcp* (coding for major capsid protein), which is usually expressed at the late stage of the viral infection cycle (29). The *P. shikokuense* relative abundance dynamics in two blooms was consistent with *psbA* expression changes, indicating that *P. shikokuense* population dynamics was closely influenced by its photosynthetic activity (Fig. 5A and B). Conversely, the trend of *mcp* expression was opposite to that of *psbA*. In the BC bloom, *mcp* expression was detected only in the morning sample when *P. shikokuense* abundance and *psbA* expression declined. In ECS, *mcp* expression decreased from T0 to T1, T2, and T3, when this species’ abundance and *psbA* expression abruptly increased, leading to a bloom outbreak.

### Characteristics of viral gene SNPs and functions of the enriched GO terms

**Fig 6:**
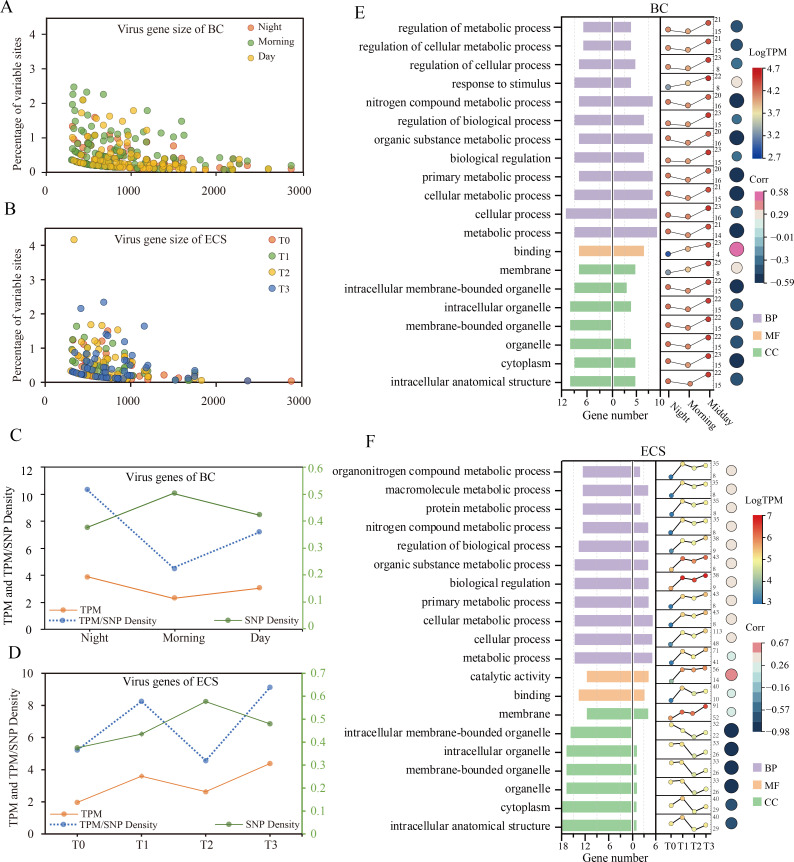
SNP profile and viral gene functions in BC and ECS blooms. (**A–B**) Inverse trends of SNP density (percent variable sites) and gene length. (**C–D**) The temporal dynamics of SNP density (Y axis on the right) and virus gene expression level (TPM, Y axis on the left), and resulting periodicity of TPM to SNP density ratio (blue line, Y axis on the left). (**E–F**) Plots showing the numbers of SNP-containing genes in the top 20 GO terms (left) and the number of differentially expressed genes in different bloom stages (right), with the X axis depicting the number of genes; The color scales on the far right, respectively, indicate the expression level data in the line plot (top), the correlation coefficient between GO terms and SNP density corresponding to the circle colors in the immediately right (middle), and categories of biological process (BP), molecular function (MF), and cellular component (CC) corresponding to histograms on the far right (bottom).

We looked for SNPs from genes of dsDNA viruses and focused on those sequenced with more than 10× depth coverage. The search yielded 456 SNPs of 108 viral genes in ECS samples and 461 SNPs of 114 virus genes in BC samples, again strikingly similar between the two blooms of the same species. We observed that smaller genes tended to have greater SNPs (Fig. 6A and B), consistent with a recent report based on long-term viral community observations (13). Besides, we noticed opposing temporal trends of expression and SNP density of these SNP-containing genes (SNPGs; Fig. 6C and D), indicating that SNP density may be inversely related to these SNPGs’ expression.

We further analyzed the top 20 GO terms of viral SNPGs in both blooms and found that they belonged to two categories (Fig. 6E and F): the composition of organelles and various metabolic processes. Comparison with differentially expressed viral genes (DEGs) indicated that SNPGs and DEGs converged in various metabolic processes in both BC and ECS. For the composition of organelle GO terms, in contrast, there were abundant SNPGs but rare DEGs. In ECS, in particular, organelles-related GO terms had no more than one DEG. As distinct membrane-bound structures, organelles in eukaryotic cells are required for various functions in viral life cycle (42).

We then documented the temporal expression changes of these organelle-related genes. An expression increase was noticed for GO terms in the BC bloom (Fig. 6E), but not in the ECS bloom. In ECS, the expression of organelle-related GO terms displayed a fluctuating but generally downward trend within a relatively small range (average 25–36 TPM), whereas metabolic-related GO terms exhibited an upward trend (average 15–45 TPM) (Fig. 6F). In addition, we found in both blooms that the expression of organelle-related functions was negatively correlated with SNP density (Fig. 6E and F), consistent with the inverse association between the bulk SNPGs expression and bulk SNP density (Fig. 6C and D). These results collectively suggest a negative regulation of organelle-related viral activity during blooms by polymorphic variants.

## DISCUSSION

This study represents the first that investigates *in situ* virus infection in dinoflagellate blooms. Our findings reveal highly similar viral assemblages that infected the bloom species *P. shikokuense* and a co-adapted metabolic relationship between the host and the viruses in the blooms, which varied between the prolonged and the short-lived blooms of the same dinoflagellate species. These findings provide new clues regarding the identity and behavior of viruses in a dinoflagellate bloom and shed light on what appears to be complex mode of infection. The novel insight will contribute to further understanding and modeling the role of viruses in regulating blooms of dinoflagellates and other algae.

Our bloom metatranscriptomes showed that dsDNA viruses from the families *Mimiviridae* and *Phycodnaviridae* dominated both blooms. This is consistent with reports to date that most dinoflagellate-infecting viruses belong to dsDNA viruses and few are single-stranded ssRNA viruses (43 – 45), and that *Mimiviridae* and *Phycodnaviridae* do infect dinoflagellates (44, 46 – 49). It is striking that even though ECS and BC are 900 km apart (Fig. 1), the taxonomic composition of dsDNA viral assemblages and the respective proportions of expressed *Mimiviridae* and *Phycodnaviridae* genes out of total virus-like genes were similar. Without electron microscopic micrographs showing viral particles in *P. shikokuense* cells, we cannot totally exclude the possibility that transcriptionally active viruses were infecting other algal species. However, our multi-angle analyses suggest that the possibility is relatively small. On the one hand, our comparative analysis of the bloom metatranscriptomes with *P. shikokuense* laboratory culture-derived transcriptomes indicated that the transcriptionally active bloom-associated viruses matched those in the *P. shikokuense* laboratory cultures, linking these viruses to the bloom species. On the other hand, we also checked if there were virus-related functions represented in our bloom metatranscriptomes from the top five abundant phytoplanktons (Fig. S3) in bloom samples. Only 6 virus-related functions (each with no more than 10 genes) were found in the other top 4 algal lineages (Fig. S3), while there are as many as 81 functions related to responses to viruses in *P. shikokuense*, including the above 6 functions. In addition, we checked whether there were any *Asfaviridae* virus genes represented in our metatranscriptomes, because the dsDNA virus that is known to infect another dinoflagellate, *Heterocapsa circularisquama*, and its DNA polymerase B gene showed high similarity to African swine fever virus (50). We only found one candidate gene (phytanoyl-CoA dioxygenase), which was retrieved from the BC bloom transcriptomes. It matched *Pacmanvirus*, which is similar to the African swine fever virus (51). We also searched for DNA polymerase genes in our metatranscriptomes and only found two sequences, which belonged to DNA polymerase A family of *Pseudomonas* phage (*Myoviridae*). These results in concert suggest that the bloom-associated viruses are likely specific to *P. shikokuense*. The detection of the ssRNA viruses in the close-shore BC bloom, their absence in the more offshore ECS bloom and cultured *P. shikokuense*, and their resemblance to mammalian viruses indicate that the potential of terrestrially sourced viruses may be transiently active and can mislead data interpretation in field work. Our finding underscores the importance to take note of the potential contamination and avoid data misinterpretation in future research.

Our finding of upregulated virus-related GO terms in the blooms suggests that both the viral life activities and host anti-virus defense were active simultaneously in host (bloom-causative) cells. The GO terms associated with virus life activities functionally covered almost all stages of the infection cycle, from attachment to release, but mostly the early stage of viral infection. From studies on various groups of algae, resistance to infection can arise at any of these stages (46). A study on strains of the dinoflagellate *Heterocapsa circularisquama* showed that resistance against viruses happened at the stage of virus nucleic acid transcription and/or replication (52). The colonial haptophyte *Phaeocystis pouchetii* achieves resistance to virus infection via the surrounding mucilage that prevents virus attachment to the cell surface (53). Another haptophyte, *Emiliania huxleyi*, escapes infection of its phycodnavirus EhV by alternating haploid and diploid stages, of which only the calcified diploid cells are susceptible to EhV (46, 54).

Although the GO term profiles pertaining to virus life activity and anti-virus defense were similar between the BC and ECS blooms, the two blooms’ chronological gene expression patterns were quite different. This potentially reflects differential infection modes between the short BC bloom and the extended ECS bloom. The elevated expression of these GO terms in the BC bloom indicates that virus life activity or anti-virus defense was heightened quickly in *P. shikokuense* cells in the one-day bloom at BC. This resembled *E. huxleyi*, in which only after 4 hours of EhV infection, infected *E. huxleyi* cells released virions (3). In another HAB species, *Aureococcus anophagefferens*, transcriptomic evidence showed that both host defense activation and viral takeover of the cell occurred as soon as 5 min post-infection (55). However, the elongation of the ECS bloom (to 20 days) coincided with the decreasing expression pattern of anti-virus defense GO terms, suggesting that the relaxation of the antiviral defense might have allowed virus infection to prolong and host cell to improve tolerance. This finding challenges the currently dominant notion that virus is an important contributor to the termination of blooms (56, 57), and viral lytic action on host cells is the major mechanism. Rather, chronic infection is not uncommon in dinoflagellates and other algae (3, 46, 49). Indeed, viruses can modify initial infection mode based on the context of the environment and host cell condition, suggesting dynamic virus-host interactions along the infection continuum (switching between lytic and non-lytic infections) (12).

The evidence of chronic viral infection of *P. shikokuense* in the ECS bloom is reinforced by the *mcp* and *psbA* expression patterns, similar to the *E. huxleyi*-EhV model (3). The *psbA* expression, the proxy of host photosynthesis and growth, showed a trend consistent with that of the relative abundance of *P. shikokuense*, indicating that the host population was healthy and active in photosynthesis and energy acquisition during the bloom, as also found in a previous study (18). Evidently, viral infection processes did not curb the growth and blooming of *P. shikokuense* in the community. In the meantime, the expression of virus budding function and host endosomal sorting complexes for transport (ESCRT) were stable throughout the ECS bloom, and these two GO terms belong to virus release function. Virus release via budding is a hallmark of chronic infections in which ESCRT plays an active role (42, 46, 58). Therefore, the activation of chronic infection-related functions in the host cells in the bloom showed that the latent temperate infection mode was likely to have allowed the establishment and maintenance of the *P. shikokuense* blooms. Conceivably, the active proliferation of the host cells in bloom is also conducive to the self-reproduction of the viruses.

Our data also suggest that virus polymorphic variants might contribute to the maintenance of chronic infection during the ECS bloom. Both blooms of the same species in the two separate ecosystems shared similar GO terms enriched by virus SNP-containing genes (SNPGs), and both showed a negative correlation between the expression of organelle-related GO terms and SNP density. While the functional connection remains to be investigated in the future, this finding implies that regulating polymorphic variants might be a genetic mechanism by which the viruses modulate organelle exploitation during infection. Previous reports indicate that viruses can manipulate and rewire host metabolic pathways to facilitate infection (59). The viruses can exploit the host cell’s important organelle and various cellular functions at different stages of their replication cycles to deliver their genome into the host cells and turn them into virus-producing units (60). Furthermore, interesting distinction was noticed in the chronological expression of SNPGs-enriched GO terms between the two blooms. Compared with the consistent upregulated expression in the BC (short-term) bloom, the expression of organelle-related function in the ECS (long-term) bloom exhibited a fluctuation around a decreasing trend, and it was more stable (fewer DEGs) than metabolic-related GO terms. This indicates that SNP may play a role in maintaining stable gene expression in organelle-related functions, which is conducive to utilizing host organelles during viral infection. Organelles are not only required for the proper functioning of cells but also for the successful infection of viruses (42). Based on the transcriptomic and SNP analyses, we posit that viruses may maintain gene expression stability in the long-term bloom through polymorphic variants, thereby sustaining their exploitation of the host organelles and maintaining chronic infection.

Host-virus interactions at microscale eventually shape ecosystem processes at geographical scales (61). Resolving the molecular mechanisms of ecologically relevant host-virus interactions is critical to understanding the role of viruses in the biogeochemical processes, as well as the factors that drive the co-evolution of virus-host systems (55). For phytoplankton-virus interactions, our current understanding is limited and research effort is scant. This study demonstrates the power of natural bloom metatranscriptomes in tandem with culture-derived transcriptomics for addressing this critical research gap. As shown in our results, this research approach allows for identifying the kind of viruses infecting the bloom algae and unveiling the functional interactions between the host and the viruses. In particular, our data indicate that chronic infection might be the dominant mode of viral infection in a prolonged dinoflagellate bloom. This provides crucial insights for future studies when relating viruses to phytoplankton bloom dynamics, in which viruses are not necessarily bloom terminators. The hypothesis derived from our data that viruses may maintain stable gene expression for crucial functions through creating SNP variants to exploit the organelles in the *P. shikokuense* sustainably and ensure the smooth progress of chronic infection warrants further investigation and critical examination. Overall, the findings and novel insight from this study will serve as the baseline for future research to fully understand and accurately model the role of viruses in regulating blooms of dinoflagellates and other algae.

## MATERIALS AND METHODS

Metatranscriptomic data were obtained using RNA-seq. Plankton samples were collected by filtering 4–13 L surface water (0–2 m) onto 3 µm polycarbonate membranes during two *P. shikokuense* blooms in 2014 at two locations that were 900 km apart (Fig. 1). The use of 3 µm filter was justified to retain nano- and micro-plankton and microbes associated with the plankton but remove free-living microbes. One of the blooms occurred in the East China Sea (ECS, 29°1′0″N, 122°9′27″E to 29°3′19″N. 122°16′30″E), and sampling was undertaken at four time points in different stages of the bloom, including the pre-bloom sample at T0 (30 April) and the bloom period samples at T1 (13 May), T2 (15 May), and T3 (20 May) as previously described (19). The other occurred at Baicheng Beach, Xiamen Harbor (BC, 24°25′ N, 118°6′ E) and was sampled three times in the bloom period, samples of 11:00 p.m. at night on 6 May and 5:00 a.m. and 1:00 p.m. on 7 May (18). Triplicate samples were collected at each sampling event. However, due to the high cost of high-throughput sequencing back in 2014, the triplicate samples from each time point were pooled for sequencing. Sequence files have been deposited at NCBI in the Sequence Read Archive under the accession number SRR8881733, SRR8881734, and SRR8881735 for Baicheng (BC) Bloom community at night, morning, and midday; SRR9878963, SRR9878964, SRR9878965, and SRR9878966 for ECS Bloom T0 to T3, respectively. A set of transcriptomic data from *P. shikokuense* cultures CCMA-206 (phosphate-replete and P-depleted condition) (22) was also used for comparison; SRR5249183 and SRR5249152 for P-depleted and P-replete (Table S4).

### Transcriptome assembly and annotation

Raw reads of mRNA sequencing were preprocessed by trimming adapter sequences and low-quality reads using SOAPnuke (version 2.1.6, parameter: -T 10 -n 0.1 -l 25 -q 0.5) (62). To retrieve all expressed genes in the bloom community, RNA-Seq reads were assembled *de novo* into contigs using MEGAHIT (version v1.2.9, parameter: --min-contig -len 200) (63). Contigs were combined and assembled into larger contigs using Tgicl (version 2.1, parameters: -l 40 -c 16 -v 25 -O ‘-repeat_stringency 0.95 -minmatch 35 -minscore 35) (64). Coding sequences (CDSs) were predicted using TransDecoder (version 5.5.0, parameter: default) (65). The function and taxonomic sources of the CDSs were annotated by searches against a local integrated database (combining MMETSP data sets, RefSeq non-redundant proteins from NCBI, and transcriptomes of *P. shikokuense* transcriptomes) (22) using Diamond (version v2.0.9, parameter: blastx -e 1e-5) (66). eggNOG (version 5.0) (67, 68) was used to obtain Gene Ontology (GO) annotations, and GO groups were obtained using clusterProfiler (69). Redundant sequences were removed from the set of CDSs using CD-HIT (version 4.8.1, parameter: -c 0.95) (70), with a sequence similarity threshold of 95%.

### Identification of viral genes

Genes with the best BLAST match to viral genes in GenBank with e-values <1e-5 were collected and named virus-like genes in this study. To associate viruses expressing these genes with the bloom dinoflagellate *P. shikokuense*, we investigated whether such viruses have been reported to infect this species or a dinoflagellate. Furthermore, the similar expression pattern of the viral genes between the two *P. shikokuense* blooms and correlation between viral gene expression pattern and the evolution of the blooms were sought and used as another supporting evidence for the functional linkage between viruses and *P. shikokuense*.

### Gene expression analysis

To explore the potential association of viruses with *P. shikokuense* cells, expression levels of virus-like (in viruses) and virus infection-related genes (in *P. shikokuense*) were measured in the BC and ECS blooms and that from *P. shikokuense* cultures (P-replete and P-depleted conditions) were used as the control group. Sequence reads were aligned back to predicted CDSs using bowtie2 (version 2.4.2) (71), and expression levels were quantified using RSEM (version v1.3.3, parameter: default) (72). Differential expression of unigenes among samples was assessed by comparing TPM (73) values of unique reads with a detection limit of ≥1 TPM. As there were no replicates in the transcriptomic data, statistical significance could not be attributed to expression data; however, any virus-related genes with expression fold change values greater than or equal to twofold or only expressed in BC and ECS blooms (and not in the pre-bloom T0 sample) were considered to be indicative of genes regulated by viruses.

### *psbA* and *mcp* identification

We used *psbA* and *mcp* as proxies of host photosynthesis and virus replication following previous reports (3, 29). *psbA* is a chloroplast gene that encodes D1 protein, involved in the first step of the light reaction during photosynthesis, and because of the rapid turnover feature of D1, cells constantly transcribe *psbA*. Viral *mcp* (coding for major capsid protein) is usually expressed at the late stage of the viral infection cycle. *psbA* sequences from functional annotation result were further checked with Photo_RC (PF00124) by hmmscan (version 3.3.2, parameters: -E 1e-5), and only proteins that matched *Prorocentrum* in taxonomy were retained; *mcp* domain file (mcp.hmm) was obtained from a previously reported work (74) and used in the same way as identifying *psbA*, and only proteins belonging to dsDNA viruses were kept.

### SNPs identification and correlation analysis

The genotype change of a species often results from the Red Queen dynamic of arms race and co-adaptation (13, 14), so we examined virus SNPs in our samples using standard tools. In brief, bowtie2 (as above) was used to map reads to virus-like genes and *P. shikokuense* genes, which were identified during the previous annotation step. The resulting alignment files were then converted from SAM to BAM format and sorted using samtools (version 1.12) (75). Variant Call Format files were created for recording variations among reads per site, which was calculated using bcftools (version 1.8, parameters: mpileup -Ou | call -vm -Oz) (75). After filtering low-quality reads and SNPs that occurred in less than 10 reads using bcftools (parameters: -e ‘%QUAL <20 || DP <10’), the remaining variants were collected a high confidence SNPs for downstream analysis. To explore the relationship between SNPs and the expression of virus genes (SNP contained) related GO terms, the correlation between genes expression of GO terms and SNP density was calculated using the R package “cor” (parameters: default) (76). Data were visualized using the R package “corrplot” (77).

## SUPPLEMENTAL MATERIAL

Supplemental figures and tables aem.01156-23-s0001.docx Fig. S1 to S4 and Tables S1 to S4

ASM does not own the copyrights to Supplemental Material that may be linked to, or accessed through, an article. The authors have granted ASM a non-exclusive, world-wide license to publish the Supplemental Material files. Please contact the corresponding author directly for reuse.
