# Supplementary material for: Active viral infection during blooms of a dinoflagellate indicates dinoflagellate-viral co-adaptation
Source: Appl Environ Microbiol. 2023 Oct 24;89(11):e01156-23. doi: 10.1128/aem.01156-23 (PMC10686096; doi:10.1128/aem.01156-23)
Supplement: Supplemental figures and tables — Fig. S1 to S4 and Tables S1 to S4 [file aem.01156-23-s0001.docx]

**Active viral infection during blooms of a dinoflagellate indicates dinoflagellate-viral co-adaptation**

Jingtian Wang^1^, Ling Li^1^, Senjie Lin

Fig. S1. Non-dsDNA virus genes of *P. shikokuense* blooms in BC and ESC.

Fig. S2. High sequence matches of viral gene sequences found in *P. shikokuense* laboratory culture transcriptome with those from the bloom metatranscriptome.

Fig. S3. Major phytoplankton-associated virus-related GO terms statistic in the two blooms. Radar chart showed the virus-related GO terms in species other than *P. shikokuense* during the two blooms. Pshi, *Prorocentrum shikokuense*; BC other, other species including *Syndiniales*, *Gonyaulacales* and *Mediophyceae*; ECS other, other species including *Karlodinium*.

Fig. S4. Enrichment of all virus-related GO terms by upregulated genes of *P. shikokuense* in the Baicheng (BC) and East China Sea (ECS) blooms.

Table S1. Sequence assembly and annotation statistics.

Table S2. Number of unigenes with nearest BLAST matches belonging to dsDNA viruses in pooled transcriptomes from each bloom event. Unique CDSs were determined using a cut-off of < 95% pairwise sequence identity. BC, Baicheng bloom; ECS, East China Sea bloom.

**Table S3**. Putative viral genes that showed increased (two-fold TPM change) expression during growing or peak bloom.

**Table S4.** Data used for comparative metatranscriptomic analysis.


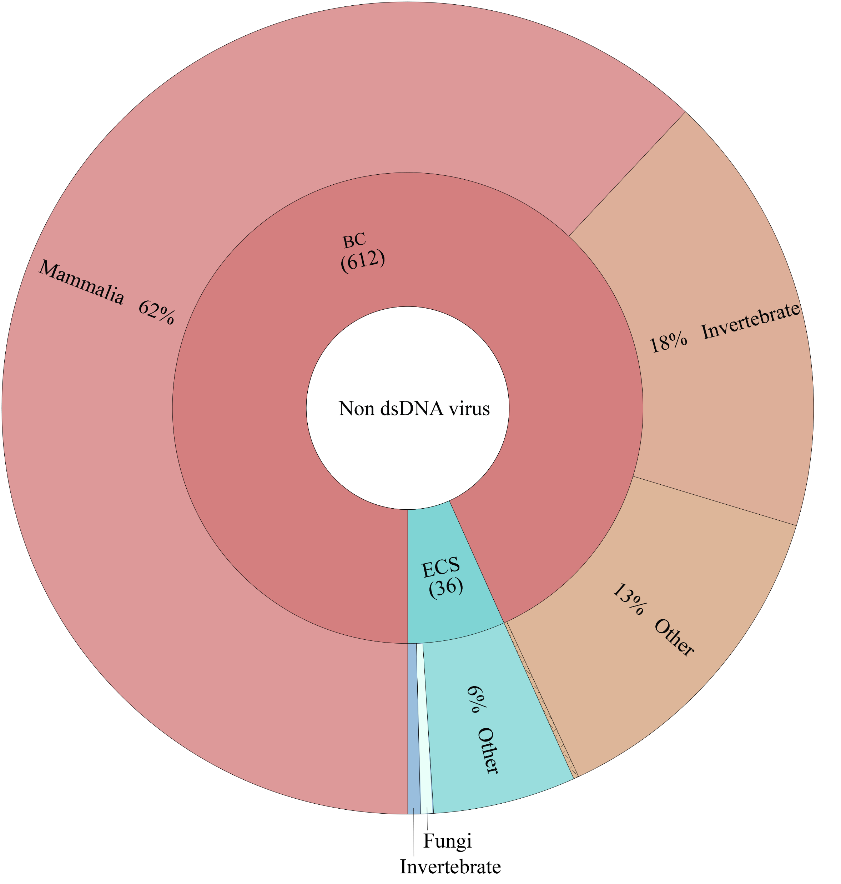


Fig. S1. Non-dsDNA virus genes of *P. shikokuense* blooms in BC and ESC.


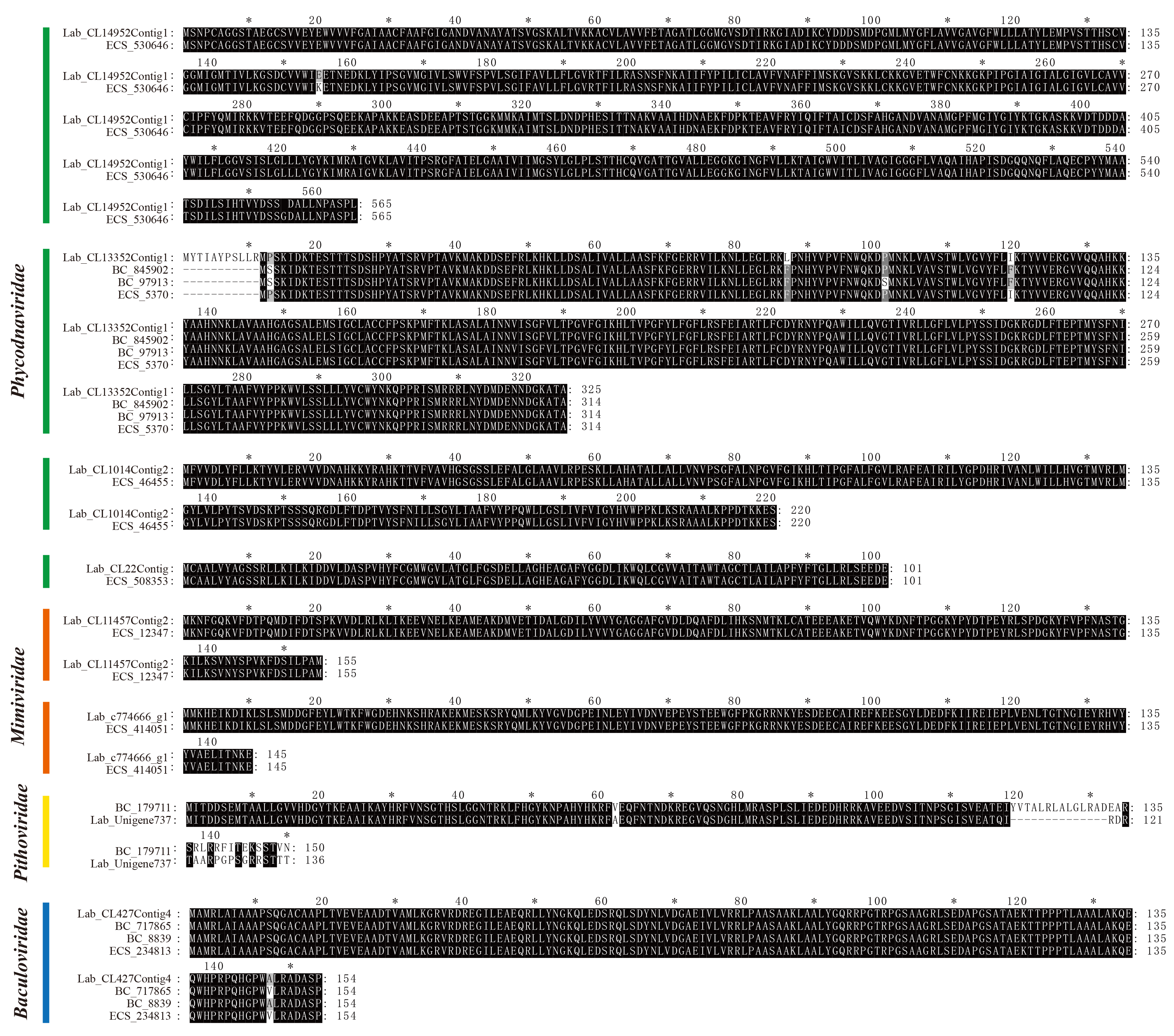


Fig. S2. High sequence matches of viral gene sequences found in *P. shikokuense* laboratory culture transcriptome with those from the bloom metatranscriptome.


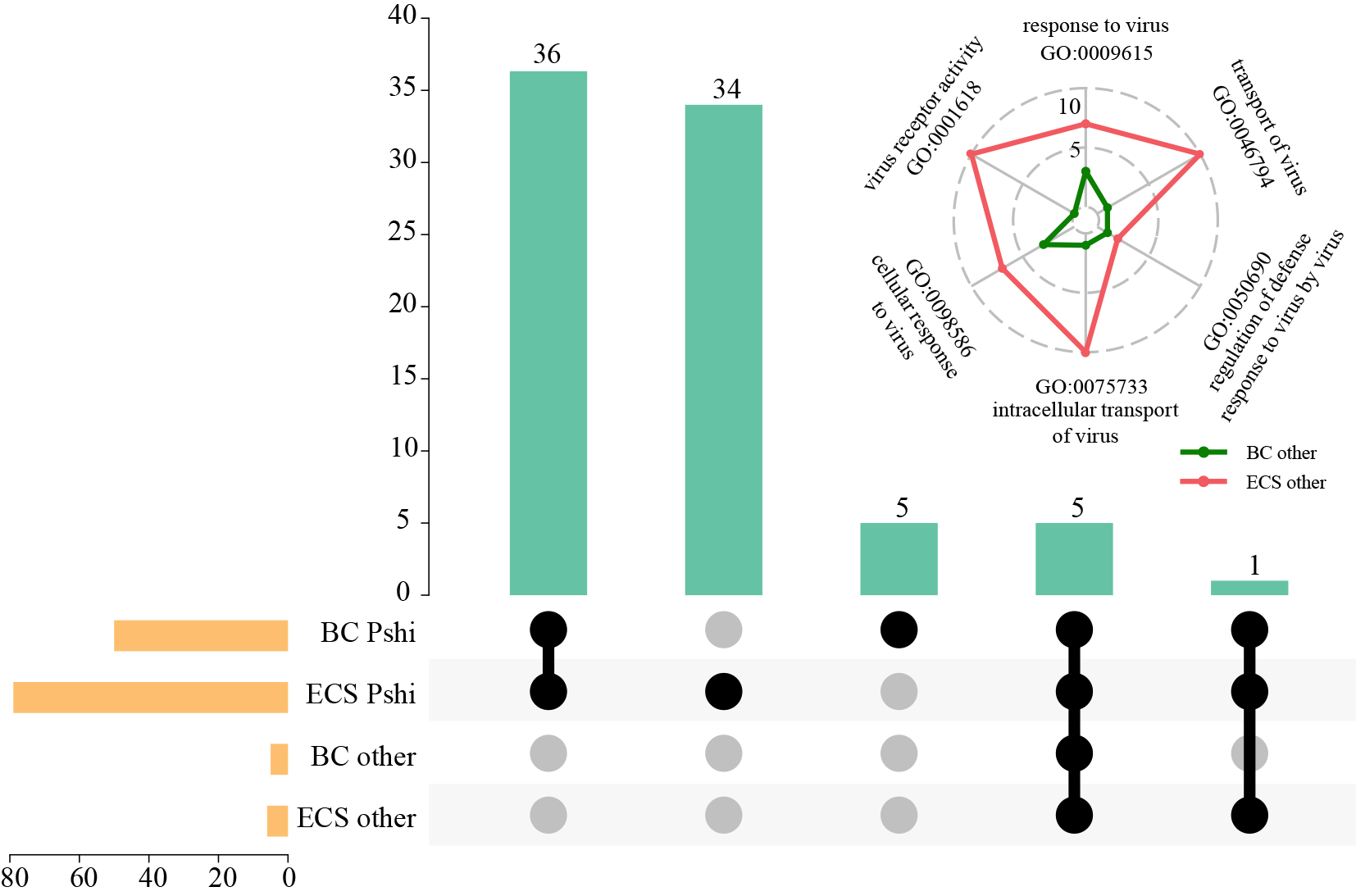


Fig. S3. Major phytoplankton-associated virus-related GO terms statistic in the two blooms. Radar chart showed the virus-related GO terms in species other than *P. shikokuense* during the two blooms. Pshi, *Prorocentrum shikokuense*; BC other, other species including *Syndiniales*, *Gonyaulacales* and *Mediophyceae*; ECS other, other species including *Karlodinium*.


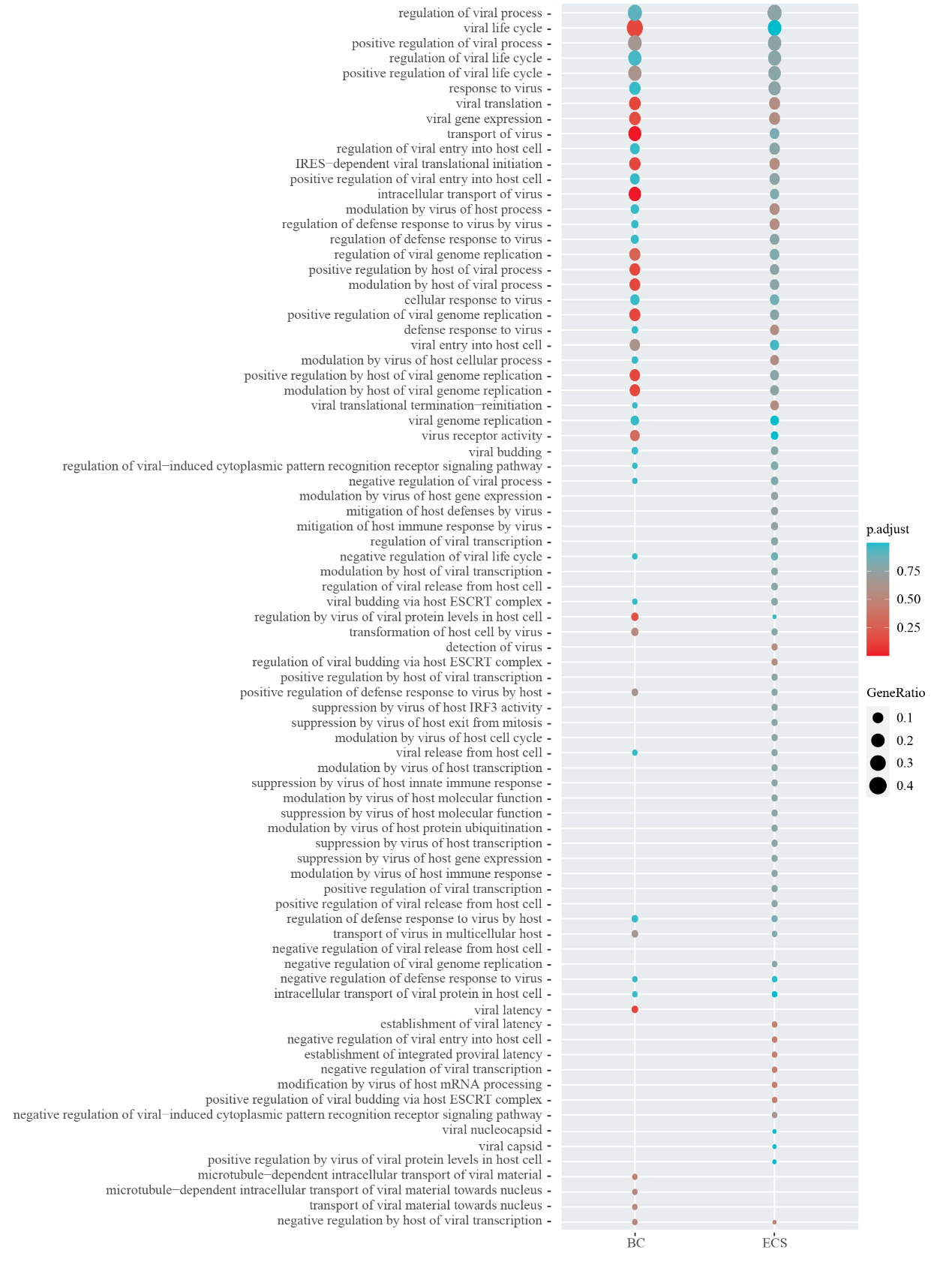


Fig. S4. Enrichment of all virus-related GO terms by upregulated genes of *P. shikokuense* in the Baicheng (BC) and East China Sea (ECS) blooms.

Table S1. Sequence assembly and annotation statistics.

|  | BC  Night | BC  Moring | BC  Midday | ECS  T0 | ECS  T1 | ECS  T2 | ECS  T3 |
| --- | --- | --- | --- | --- | --- | --- | --- |
| ASSEMBLY No. of contigs | 228,030 | 756,308 | 236,386 | 319,719 | 263,147 | 239,849 | 138,182 |
| Mean contig length (bp) | 876 | 607 | 686 | 587 | 542 | 552 | 551 |
| GC (%) | 56.57 | 48.19 | 54.44 | 49.34 | 55.12 | 52.52 | 54.18 |
| N50 (bp) | 1,177 | 636 | 755 | 615 | 553 | 570 | 578 |
| ANNOTATION No. of predicted CDS | 158,866 | 536,568 | 113,914 | 254,500 | 202,659 | 162,735 | 84,559 |
| No. of non-redundant genes | 151,765 | 532,418 | 111,093 | 253,245 | 201,790 | 162,102 | 84,026 |
| non-redundant CDSs with BLASTX matches | 91,634 | 360,284 | 77,285 | 173,894 | 120,849 | 94,878 | 47,958 |
| No. of non-redundant virus-like genes* | 97 | 863 | 71 | 198 | 84 | 67 | 141 |

Table S2. Number of unigenes with nearest BLAST matches belonging to dsDNA viruses in pooled transcriptomes from each bloom event. Unique CDSs were determined using a cut-off of < 95% pairwise sequence identity. BC, Baicheng bloom; ECS, East China Sea bloom.

| **dsDNA virus** | **BC** | **ECS** |
| --- | --- | --- |
| *Mimiviridae* | 272 | 160 |
| *Phycodnaviridae* | 112 | 68 |
| *Pithoviridae* | 68 | 15 |
| *Marseilleviridae* | 29 | 15 |
| *Baculoviridae* | 11 | 3 |
| *Iridoviridae* | 3 | 5 |
| *Poxviridae* | 3 | 1 |
| *Medusaviridae* | 3 | 0 |
| *Podoviridae* | 1 | 1 |
| *Pandoraviridae* | 1 | 1 |
| *Asfarviridae* | 1 | 0 |
| *Adintoviridae* | 0 | 1 |
| *other* | 67 | 127 |
| sum | 571 | 397 |

**Table S3. Putative viral genes that showed increased (two-fold TPM change) expression during growing or peak bloom.**

| **Bloom site** | **Description** | **Neatest BLASTX match** | **Accession number** | **E-value** | **Percentage of match (%)** |
| --- | --- | --- | --- | --- | --- |
| ECS | hypothetical protein EXVG_00204 | *Emiliania huxleyi* virus 202 | AET42553.1 | 8.34E-79 | 50.6 |
| ECS | hypothetical protein EXVG_00204 | *Emiliania huxleyi* virus 202 | AET42553.1 | 1.49E-74 | 49 |
| ECS | phosphoribosyl-ATP pyrophosphohydrolase | *Edafosvirus* sp. | AYV78335.1 | 2.16E-56 | 57.6 |
| ECS | phosphoribosyl-ATP pyrophosphohydrolase | *Edafosvirus* sp. | AYV78335.1 | 4.04E-58 | 58 |
| ECS | fatty acid elongase | *Emiliania huxleyi* virus 202 | AET42571.1 | 3.78E-68 | 48.6 |
| ECS | phytanoyl-CoA dioxygenase | *Fadolivirus* 1 | QKF94656.1 | 3.98E-76 | 40.9 |
| ECS | 23-sialyltransferase | NY_014 *poxvirus* | YP_009408543.1 | 6.04E-22 | 30.2 |
| ECS | ubiquitin-conjugating enzyme E-17 kDa 3 | *Terrestrivirus* sp. | AYV75765.1 | 1.10E-34 | 40.9 |
| ECS | hypothetical protein HWQ62_00392 | *Pyramimonas orientalis* virus | QOI90527.1 | 3.25E-22 | 28.1 |
| ECS | mimivirus elongation factor aef-2 | *Tupanvirus* deep ocean | QKU34624.1 | 1.77E-48 | 37.2 |
| ECS | cytidylyltransferase-like protein | *Marseillevirus* LCMAC102 | QBK86642.1 | 3.57E-15 | 33 |
| ECS | inter-alpha-trypsin inhibitor family protein | *Fadolivirus* 1 | QKF93464.1 | 1.76E-23 | 28.8 |
| ECS | hypothetical protein Terrestrivirus | *Terrestrivirus* sp. | AYV75154.1 | 2.19E-23 | 39.7 |
| BC | hypothetical protein YASMINEVIRUS_128 | *Yasminevirus* sp. GU-2018 | VBB17665.1 | 2.12E-41 | 53.6 |
| BC | U-box and intein containing von Willebrand factor type A | *Chrysochromulina ericina* virus | YP_009173706.1 | 4.38E-50 | 33.9 |
| BC | putative cytidine deaminase | *Acanthamoeba polyphaga mimivirus* | YP_003986693.1 | 2.99E-07 | 41.1 |
| BC | putative envelope protein | *Escherichia* virus Lambda | NP_597781.1 | 2.50E-114 | 99.4 |
| BC | putative eukaryotic translation initiation factor 5A-1 | *Harvfovirus* sp. | AYV81692.1 | 1.55E-13 | 32.8 |
| BC | fatty acid elongase | *Emiliania huxleyi* virus 202 | AET42571.1 | 1.65E-68 | 41.7 |
| BC | glycosyltransferase | *Yasminevirus* sp. | VBB18282.1 | 1.59E-09 | 26.4 |
| BC | major capsid protein | *Cafeteria roenbergensis* virus BV-PW1 | YP_003969975.1 | 2.30E-172 | 55.4 |
| BC | putative mannosyltransferase | *Lausannevirus* | YP_004347014.1 | 1.23E-11 | 36.1 |
| BC | mitochondrial carrier protein | *Fadolivirus* 1 | QKF93583.1 | 6.20E-45 | 59.3 |
| BC | mitochondrial carrier protein | *Fadolivirus* 2 | QKF93583.1 | 2.11E-79 | 55.9 |
| BC | hypothetical protein EXVG_00204 | *Emiliania huxleyi* virus 202 | AET42553.1 | 4.68E-74 | 49 |
| BC | hypothetical protein EXVG_00205 | *Emiliania huxleyi* virus 203 | AET42553.1 | 1.34E-78 | 50.6 |
| BC | hypothetical protein EXVG_00206 | *Emiliania huxleyi* virus 204 | AET42553.1 | 6.44E-70 | 50.4 |
| BC | hypothetical protein Hyperionvirus21_15 | *Hyperionvirus* sp. | AYV84281.1 | 1.29E-17 | 26.9 |
| BC | oxidoreductase domain containing protein | *Yasminevirus* sp. | VBB18170.1 | 5.46E-40 | 36.1 |
| BC | phosphoribosyl-ATP pyrophosphohydrolase | *Edafosvirus* sp. | AYV78335.1 | 3.45E-56 | 57.6 |
| BC | hypothetical protein | *Tupanvirus* soda lake | QKU35374.1 | 7.91E-40 | 43.8 |
| BC | hypothetical protein Solumvirus2_34 | *Solumvirus* sp. | AYV86227.1 | 7.75E-41 | 51.9 |
| BC | hypothetical protein HWQ62_00392 | *Pyramimonas orientalis* virus | QOI90527.1 | 5.53E-24 | 31.6 |
| BC | hypothetical protein HWQ62_00393 | *Pyramimonas orientalis* virus | QOI90527.1 | 9.97E-19 | 26.2 |
| BC | hypothetical protein YASMINEVIRUS_128 | *Yasminevirus* sp. | VBB17665.1 | 1.97E-28 | 57.3 |
| BC | core protein | *Yasminevirus* sp. | VBB18083.1 | 1.45E-246 | 59.7 |

**Table S4. Data used for comparative metatranscriptomic analysis.**

| **Sample** | **Source** | **Condition** | ***P. shikokuense* fraction in Bloom (%)** | **Data volume**  **(Gpb)** | **SRA** |
| --- | --- | --- | --- | --- | --- |
| T0 | East China Sea (ECS) | Pre/non-bloom (9:25,30th April 2014) | 4.0 | 8.56 | SRR9878963 |
| T1 |  | Bloom (11:40,13th May 2014) | 54.0 | 10.92 | SRR9878964 |
| T2 |  | Bloom (11:40,15th May 2014) | 50.5 | 10.72 | SRR9878965 |
| T3 |  | Bloom (15:00,20th May 2014) | 80.3 | 11.89 | SRR9878966 |
| Night | Baicheng (BC) | Bloom (23:00,6th May 2014) | 92.8 | 15.60 | SRR8881733 |
| Moring |  | Bloom (5:00,7th May 2014) | 50.2 | 14.30 | SRR8881734 |
| Midday |  | Bloom (13:00,7th May 2014) | 85.5 | 16.60 | SRR8881735 |
| P repleted (Normal) | Culture | L1 medium with 36 μM P | - | 12.40 | SRR5249152 |
| P depleted |  | L1 medium with 1 μM P | - | 12.10 | SRR5249183 |
